# Lymphopenia assessment in ICU patients and relationship with mortality

**DOI:** 10.1186/2197-425X-3-S1-A340

**Published:** 2015-10-01

**Authors:** R Gutierrez-Rodriguez, E Aguilar-Alonso, MD Arias-Verdu, E Castillo-Lorente, C Lopez-Caler, M Rojas-Amezcua, C De la Fuente-Martos, R Rivera-Fernandez, G Quesada-Garcia

**Affiliations:** Intensive Care, Regional University Hospital, Malaga, Spain; Intensive Care, Infanta Margarita Hospital, Cabra, Spain; Intensive Care, Hospital Neurotraumatologico, Jaen, Spain

## Introduction

White blood cell count is a parameter that is included in many scoring systems. Previous studies based on acute coronary syndrome are demonstrated that the ratio of neutrophils to lymphocytes is related to mortality. The relationship between lymphocytes count and mortality is unknown in critically ill patients.

## Objectives

To assess the relationship between ICU mortality and disturbance in the lymphocytes count.

## Methods

Multicenter case-control study nested in a cohort. Patients hospitalized to a period of four months in the ICU of the two hospital (University Clinical -Malaga- and Infanta Margarita- Cabra, Córdoba-) are included. We have studied all deaths and a random sample (1 of 5) of the survivors.

## Results

226 patients (178 Malaga, 48 Cabra). 89 cases (deaths). And 138 controls that are the random sample of survivors. Mean age was 63.39 ± 14.04 years. The severity assessed by SAPS-3 in 48.84 ± 15.39 points.

The deaths present at admission leukocytes count 13614 ± 6137 vs. 10212 ± 4759 of the controls (p < 0.001). The deaths also present on admission greater neutrophils count 10813 ± 6258 vs 7823 ± 4396 (p < 0.001) and lower lymphocytes count 1194 ± 826 vs 1471 ± 1355 (p < 0.001). The ratio of neutrophils and lymphocytes increased in deaths (15.55 ± 24.14 vs 8.38 ± 7.54, p < 0.001).

We collect the lowest lymphocytes count throughout all ICU stay and the deaths have lower lymphocyte count that survivors (768 ± 494 vs 1120 ± 893, p < 0.001). Multivariate analysis showed that variable most related with mortality is the lowest lymphocytes count. The 47.2% of deaths patients had lymphocytes count less that 600 in the analytical with lower lymphocyte count, compared to 23.4% of the survivors (p < 0.001), OR: 2.93 (1.65-5.21).

## Conclusions

The mortality of ICU patients is associated with leukocytosis, neutrophilia, lymphopenia and high ratio of neutrophils and lymphocytes. The deaths usually have very low lymphocytes count.
